# The effect of aging in primary human dermal fibroblasts

**DOI:** 10.1371/journal.pone.0219165

**Published:** 2019-07-03

**Authors:** Juliana Carvalhães Lago, Maria Beatriz Puzzi

**Affiliations:** Department of Dermatology, School of Medical Sciences, Laboratory of Skin Cell Cultures-Pediatric Research Center, University of Campinas – UNICAMP, Campinas, São Paulo, Brazil; San Gallicano Dermatologic Institute, ITALY

## Abstract

Skin aging is a complex process, and alterations in human skin due to aging have distinct characteristic as compared to other organs. The aging of dermal cells and the biological mechanisms involved in this process are key areas to understand skin aging. A large number of biological mechanisms, such as decreasing of protein synthesis of extracellular matrix or increasing of degradation, are known to be altered through skin aging. However, environmental influence can accelerate this characteristic phenotype. In this study, we analyzed primary human dermal fibroblasts in three different in-vitro aging models—UVB irradiation and accelerated proliferation of human dermal fibroblasts from young donors as well as from elderly donors—for the gene expression of *COL1A1*, *COL1A2*, *COL3A1*, *COL4A1*, *COL7A1*, *MMP1*, *MMP2*, *MMP3*, *MMP7*, *MMP8*, *MMP9*, *MMP10*, *MMP12*, *MMP13*, *MMP14*, *TIMP1*, *TIMP2*, *TIMP3*, *TIMP4*, *IL1B*, *IL1A*, *IL6*, *IL8*, *IL10*, *PTGS2*, *TP53*, *CASP3*, *LMNA*, *SIRT1*. We compared the gene expression levels with young control. Furthermore, the behavior of skin fibroblasts was also evaluated using cell growth rate. The findings reveal that the gene expression levels in skin fibroblasts was altered in the process of aging in all three in-vitro aging models, and the cell growth rate was reduced, suggesting that these methods can be employed to understand skin aging mechanisms as well as drug discovery screening method.

## Introduction

The skin is the most exposed organ of the body, and it functions as a barrier against external aggressions. It frequently experiences the direct effects of environmental exposure, including UV radiation and air pollution [1]. Alterations in skin structure and physiology occur as natural consequences of aging and contribute to diminished cutaneous health [2; 3; 4]. These damages can be aggravated by external factors and, combined with lifestyle, result in significant biological alterations, characteristic of premature aging [5; 6; 7]. The mechanisms to accelerate skin aging include increase of reactive oxygen species (ROS), mtDNA mutations, and telomere shortening as well as hormonal changes [8; 9] produced by excessive exposure to environmental factors. Extrinsic aging, influenced by environmental factors, varies among individuals and ethnic groups in a manner different from the natural aging [6].

Exposition to high levels of UV radiation is a major cause for oxidative stress, resulting in differential expressions of endogenous antioxidant enzymes, proteins oxidation and lipid peroxidation. Both the dermis and epidermis are affected by sunlight and UV radiation exposure [10; 11]. Skin cells exhibit complex alterations during aging and are frequently accompanied or caused by changes in gene expression and the susceptibility of cell transformation [12; 13], related to extracellular matrix proteins [14; 15; 16]. The areas exposed to the sunlight display, in general, deep and fine lines, wrinkles, rough and dry skin, alterations in skin pigmentation as dark spots, sagging, and tensile strength loss [17; 18].

The matrix metalloproteinases (*MMPs*) play an important role in tumor invasion, inflammation, and skin aging [19]. Natural aging and premature aging, induced by UV radiation, invokes the expression of MMPs that degrades dermal collagen and other proteins from the extracellular matrix [20; 21; 22], thus resulting in wrinkles and sagging. Studies have also indicated the importance of TIMPs, known as metalloproteinase inhibitors, which play a significant role in the inhibition of protein degradation of the extracellular matrix [23; 24].

The UVB irradiation induces inflammatory cytokines expression and contributes to acute and chronic cutaneous damages through inflammatory mediators generated by ROSs common in the aging process [11; 19; 25]. *CASP3* plays a central role in the apoptosis pathway and is described as an important marker in studies with UVR exposition and photoaging [26; 27]. Another gene related to the physiological process of cellular aging is *SIRT1*, which plays an important role in maintaining longevity [28].

Mutations in *LMNA* are associated to several diseases, including Hutchinson-Gilford progeria syndrome (HGPS), a premature aging disorder in which individuals present with senile diseases specific to the aging process, as well as dermatosis [29; 30]. The mutation results in the expression of a truncated form of Lamin A, called progerin, whose accumulation has not only been described in HGPS but also during normal and photo-stimulated aging [31].

In this study, we evaluate the genes involved in the skin aging process in terms of intrinsic and extrinsic aging, to verify the gene expression profile of aging. Furthermore, we also evaluate the viability of these genes as aging markers for in-vitro models pertaining to aging studies or drug discovery screening that can provide pre-clinical solutions for numerous age-related disturbances in the skin caused by extrinsic and intrinsic factors.

## Material and methods

### Statement of ethics

Primary human dermal fibroblasts (HDF) from six healthy, young, male and five healthy, elderly, female donors were collected through posthectomy performed by the pediatrics surgery group and blepharoplasty performed by the ophthalmic plastic group of the School of Medical Sciences at UNICAMP respectively. Moreover, the use of these samples has been allowed in the research project FR378403, approved by the Ethics Committee of FCM/UNICAMP on 26/01/2011.

Individuals over 18 years of age provided their written informed consent for their participation in this study and signed the ICF (informed consent) in accordance with Resolution 196/96 and approved by the FCM/UNICAMP Ethics Committee.

For individuals under 18 years old, their legal guardians provided the written informed consent for their participation in this study and signed the ICF (informed consent) in accordance with Resolution 196/96 and approved by the FCM/UNICAMP Ethics Committee.

### Isolation of cells

Primary HDF from young healthy donors, less than 10 years old, and from elderly healthy donors, over 55 years old, were cut into several fragments of approximately 5mm^2^ and stored in 5mL trypsin (INVITROGEN) separately in sterile Petri dishes to separate the dermis from epidermis. After 4 hours, 5mL M199 medium (GIBCO) + 10% fetal bovine serum (NUTRICELL) were added for trypsin neutralization, and the result was centrifuged for 5 minutes at 2000 rpm [32; 33]. Next, the dermis was transferred to a cell culture flask with 10mL medium M199 + 10% FBS and kept in it for at least 24 hours. Fibroblasts from the dermis were proliferated until passage 5, and after that, the RNA was extracted.

### UVB irradiation of fibroblast

Primary HDF from young healthy donors, passage 5, were submitted to a subcytotoxic dose of 1 J/cm^2^ UVB radiation in four series of 0.25 J/cm^2^ radiation at 24-hour intervals, utilizing Bio-Sun (Vilber Lourmat) and Hanks culture medium (SIGMA) without FBS. After 24 hours of the last irradiation, the mRNA was extracted. This subcytotoxic dose exposed several biomarkers of senescence [34].

### Accelerated proliferation of fibroblast

The primary HDF from young healthy donors were proliferated using trypsin (INVITROGEN) until passage 20, and the reduction of cell growth rate was observed [35; 36]. Subsequently, the mRNA was extracted.

### Cell senescence profile

All the cells from this study were evaluated for cell senescence profile employing the senescence-associated β-galactosidase activity technique summarized here and the cell proliferation time technique, using Scepter Cell Counter and electric cell-substrate impedance sensing ECIS.

The cellular senescence of HDF from young donors exposed to UVB irradiation and accelerated proliferation as well as HDF from elderly donors and the young HDF control were measured via staining with X-gal (5-bromo-4-chloro-3-indolyl-beta-d-galactopyranoside) at pH 6.0, a condition that suppresses the lysosomal beta-galactosidase activity to guarantee that most non-senescent cells will appear unstained [37].

For the cell proliferation time technique, HDF from each donor were cultivated in 6 well plates with 2x10^4^ cells per well in the M199 culture medium + 10% BFS at 37°C and 5% CO^2^; this was repeated with all the HDF cultures. After 72 hours (3 days), the cells from 3 wells were trypsinized, mixed in a pool and counted using the Scepter Cell Counter (Merck Millipore). This protocol was repeated after 144 hours (day 6) with the remaining 3 wells [S1 File].

For cell culture specific to ECIS equipment use, HDF were cultivated on an adapted array with 1x10^4^ cells/well in M199 culture medium + 10% BFS at 37°C and 5% CO^2^. The same process was repeated with all the HDF cultures. The M199 + 10% SFB culture medium was added to a cell-free well as a control. Real-time cell proliferation analysis was performed according to the ECIS protocol [38] for 60 hours at the frequency of 4000 Hz. The result was determined in terms of resistance (Ohm) for each donor [S2 File].

### Gene expression analysis

The mRNA from the HDF was extracted using the RNeasy mini kit (Qiagen) according to the manufacturer’s instructions [39]. The cDNA was synthesized utilizing a High-Capacity cDNA Reverse Transcription Kit (Applied Biosystems), and a real-time RT-PCR analysis was performed according to the Taqman protocol (Applied Biosystems) using the Taqman assay and Thermal Cycler Rotor-Gene 3000 (CORBETT RESEARCH—QIAGEN).

The genes analyzed were sorted according to their influence on the aging process: *COL1A1*, *COL1A2*, *COL3A1*, *COL4A1*, and *COL7A1* are responsible for collagen synthesis; *MMP1*, *MMP2*, *MMP3*, *MMP7*, *MMP8*, *MMP9*, *MMP10*, *MMP12*, *MMP13*, and *MMP14* degrade proteins of the extracellular matrix; *TIMP1*, *TIMP2*, *TIMP3*, and *TIMP4* inhibit the metalloproteinase action; *IL1B*, *IL1A*, *IL6*, *IL8*, *IL10*, and *PTGS2* participate in the inflammatory pathway and play an important role in the process of aging and cutaneous damage; *TP53* is an important marker for cell division; *CASP3* is central to the apoptosis process; *LMNA* is related to premature aging; *SIRT1* is an important marker for longevity and aging studies. Gene expression levels were estimated using the ΔΔCt methodology [40] and keeping HDF from young donors as a control [S3 File].

### Statistical analysis

The statistical significance between the three in-vitro models groups using the ΔΔCt results from the real-time PCR assay was obtained by performing ANOVA in the XLSTAT 2007 program, p<0.05. For the Scepter Cell Counter assay, this was analyzed by Test Z, XLSTAT 2007 program, p<0.0001 [S4 File].

## Results

In this study, we analyzed primary HDF from young healthy donors as well as three different in-vitro aging models—UVB irradiation and accelerated proliferation of HDF from young donors as well as from elderly donors—with regard skin aging characteristics: cell senescence, cell growth rate and gene expression profile.

### In-vitro aging models

#### Senescence-associated β-galactosidase activity

**Fig 1:**
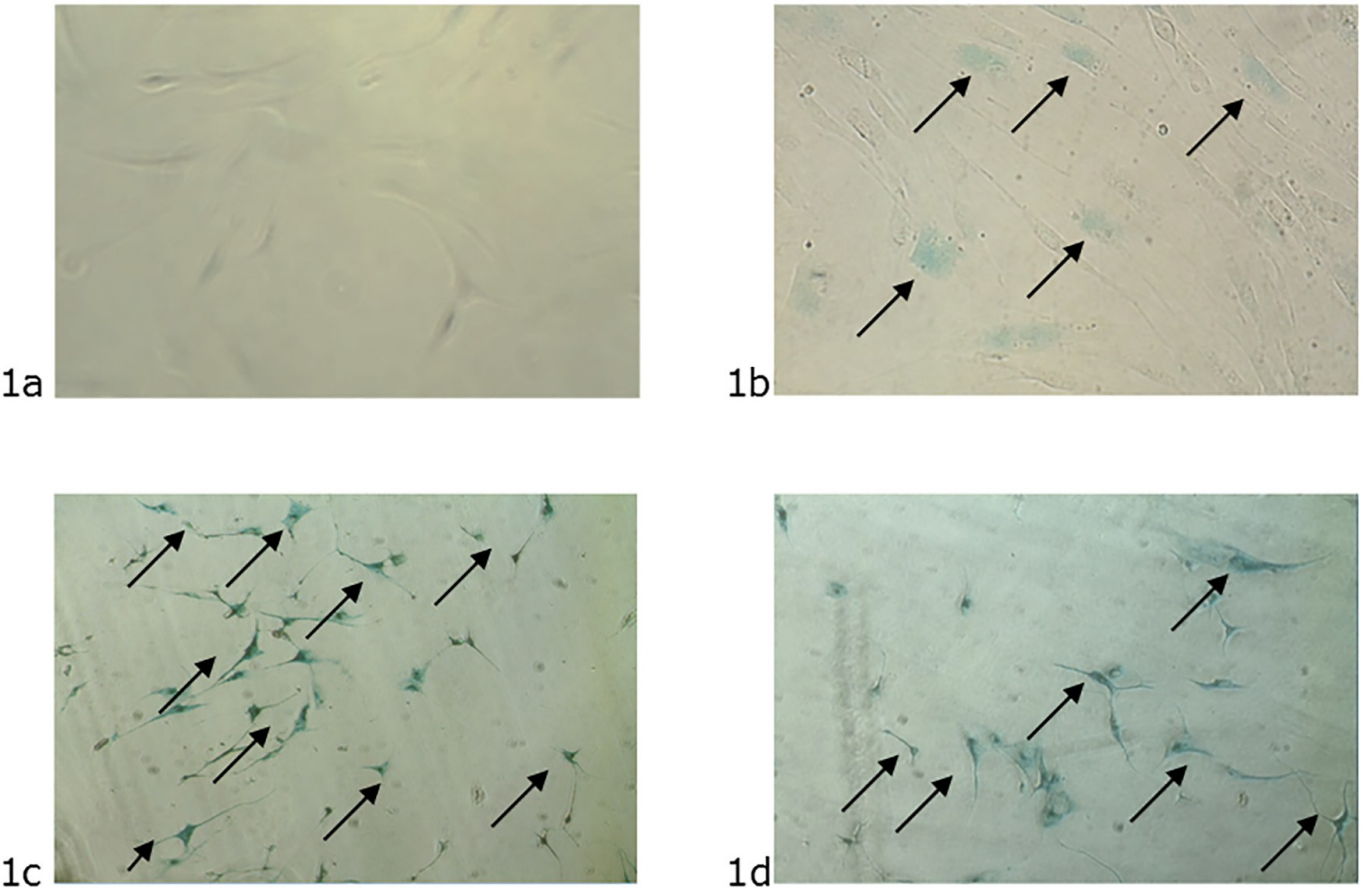
Images from premature senescence activity induced by stress. 1a –Primary HDF from the young donors control assay at passage 5. The absence of the blue color indicates cells with non-senescence activity. 1b –Primary HDF from elderly donors at passage 5. The blue color indicates a few senescent cells. 1c –Primary HDF from young donors submitted to UVB irradiation at passage 5. The blue color indicates cells with senescence activity. 1d –Primary HDF from young donors submitted to accelerated proliferation at passage 20. The blue color indicates cells with senescence activity.

Here, we presented four representative images from each HDF culture, demonstrating the senescence activity related to aging. The blue color from cells indicates activity of β-galactosidase enzyme reacting with the X-GAL dye [37]. The premature senescence of the HDF is characterized by a large number of cells in the senescence activity, exhibited by the strong blue coloration (Fig 1c and 1d). The HDF from elderly donors (Fig 1b) presented a few senescent cells, but there were cells with senescence activity. It was not possible to observe senescence activity in the cells from young donors (Fig 1a).

#### Scepter cell counter assay

Senescence is characterized by alterations in the physiology and morphology of cells beyond only diminished cell proliferation rate [35; 36; 41; 42; 43; 44]. We performed a cell-counting assay to confirm the senescence phenotype of aged HDF. All the cells groups were initially cultivated with 2x10^4^ cells per well.

**Fig 2:**
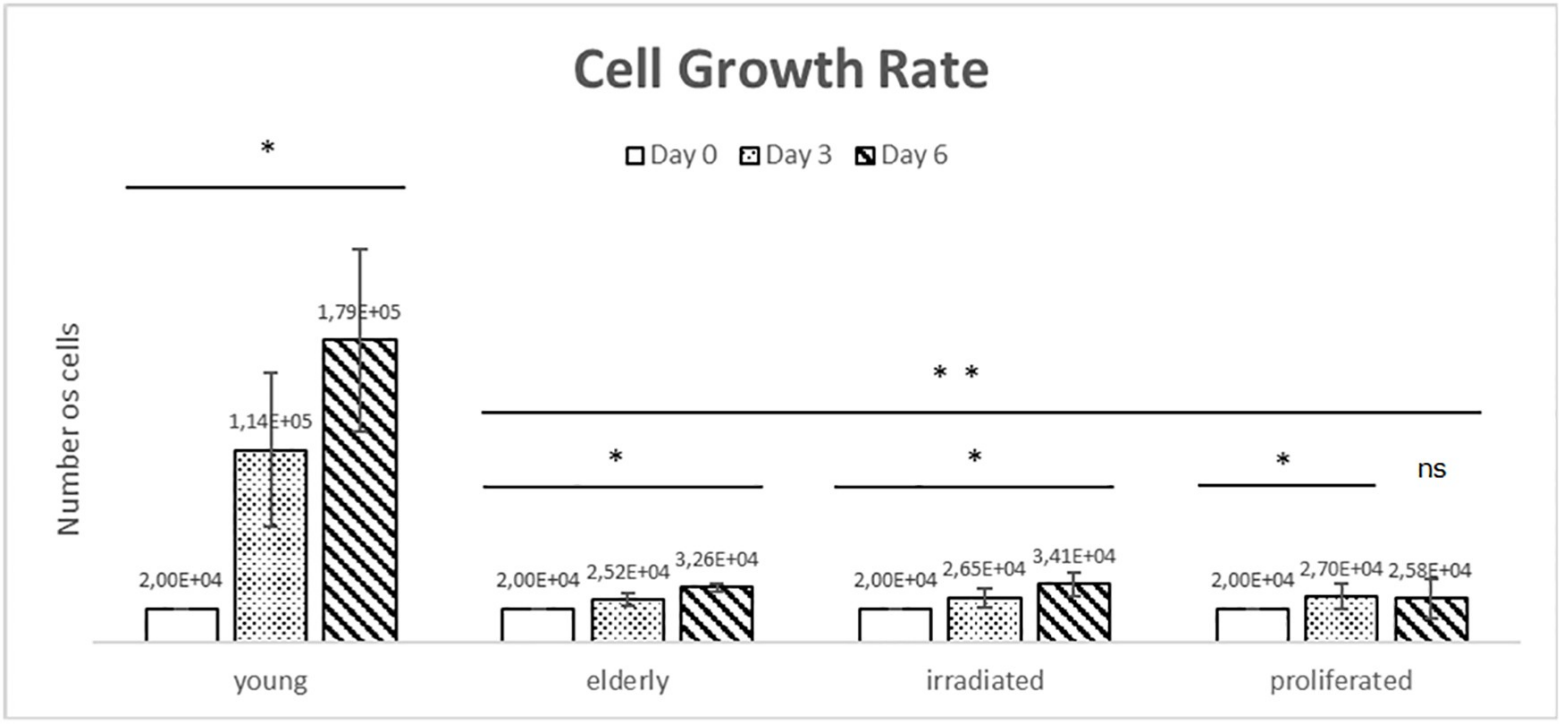
Cell growth rate. The HDF cell growth rates from all the in-vitro aging models were analyzed after 3 and 6 days of incubation. Data is expressed in number of cells, and the results were compared between days 0, 3, and 6 (*) and between young HDF donors versus aged cells (**). Test Z statistics (p<0.0001; ns: not significant).

We observed decreased cell growth rate in all three in-vitro aging models with regard to the HDF from young donors (Fig 2). The number of cells from HDF young donors on day 3 and 6 was 1.14x10^5^ and 1.79x10^5^ respectively (Fig 2). This result represents a significant increase in cell growth rate compared to day 0. The cell growth rates of the HDF from elderly donors from day 3 and day 6 were 2.52x10^4^ and 3.26x10^4^ respectively, indicating a substantial increase in proliferation capacity compared to day 0. Moreover, the HDF under UVB irradiation saw an increase in cell growth rate to 2.65x10^4^ and 3.41x10^4^ for days 3 and 6 respectively (Fig 2). The accelerated proliferation of HDF presented cell growth rates of 2.70x10^4^ and 2.58x10^4^ on day 3 and 6 respectively, which was no significance increase compared to day 0 (Fig 2). These findings are associated with literature prerogatives [42; 43].

#### Electric cell-substrate impedance sensing ECIS assay

**Fig 3:**
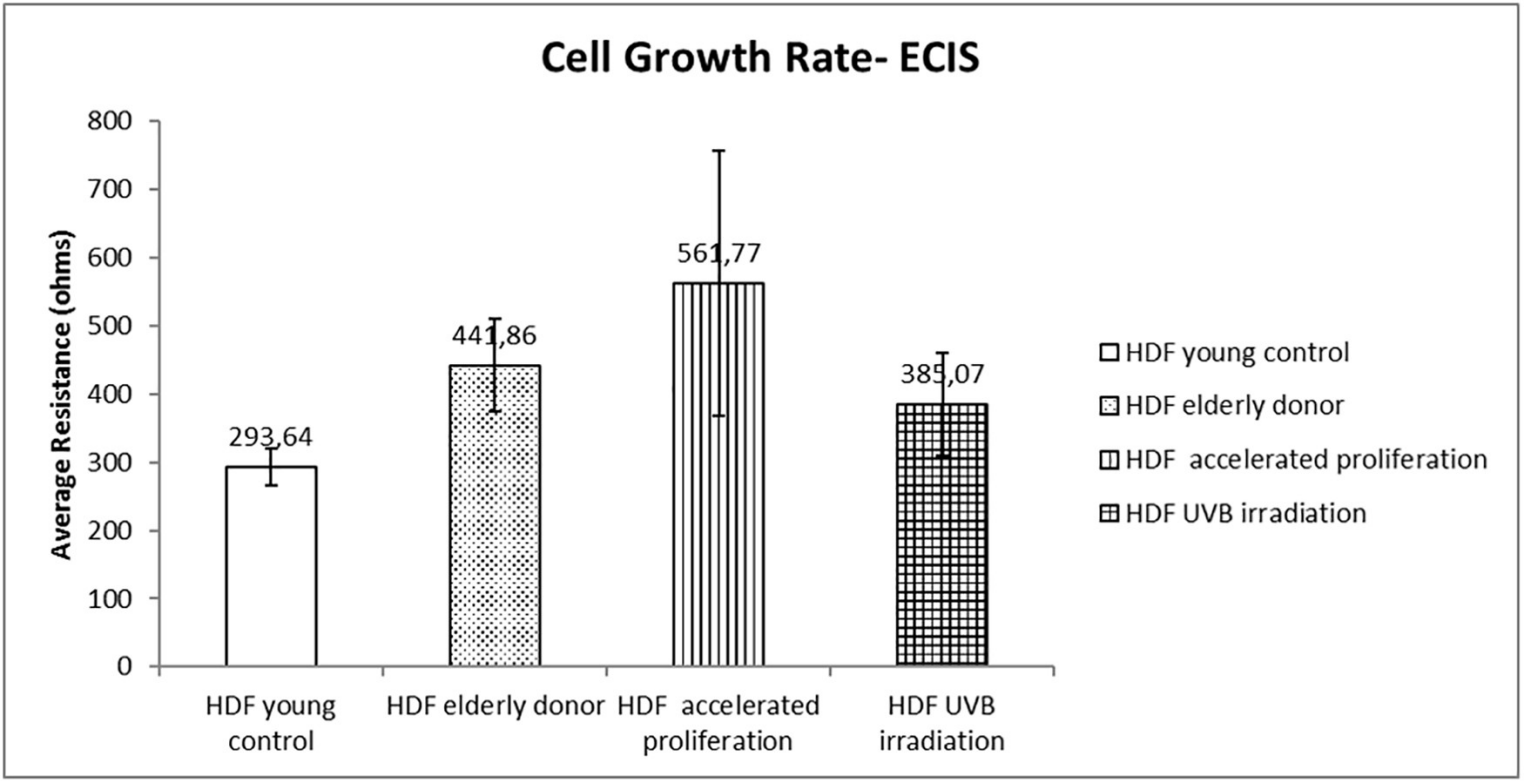
Cell growth rate: Real-time evaluation by resistance and impedance measurement with ECIS (electric cell-substrate impedance sensing). The analysis of the cell growth rate, evaluated in real time by determining the resistance in relation to time, obtained via impedance sensing indicates an increase in impedance in comparison to young control.

We evaluated the cell growth rate in this second assay utilizing a real-time methodology to identify the doubling time of the cell culture via impedance sensing. At the 35th hour of the experiment, we observe an average impedance as presented in Fig 3.

The applied electric field produces a voltage drop at the boundary between the solution and electrode. The cells that attach and spread on the electrode have an effect on the measured impedance, and that can also cause it to fluctuate with time [45; 46]. The impedance behavior indicates diminished time for in-vitro aged HDF to fill the space of the well during cell growth as compared to HDF from young donors, which is in contrast with cell counter assay results. These finds reinforce a well-documented senescence phenotype that include alterations in cell morphology during aging [47; 48], which will be discussed below.

### Gene expression analysis

The gene expression assay was performed by comparing the HDF from all in-vitro aging models to determine dermal aging profile, considering the HDF from young donors as the control using the ΔΔCt method [40]. The ratio was converted into fold change and grouped according to the upregulation or downregulation profile [Table 1]. This result suggests aging-related differential gene expression in all three in-vitro models. However, the expression of the genes was not uniform between in-vitro models. The dermal aging phenotype may vary with external stimuli, such as UV radiation or cell culture conditions [10; 49].

**Table 1:** Gene expression profile using young HDF donors as the control.

*Symbol*	*Entrez gene name*	*Location*	*UVB irradiation*	*Accelerated proliferation*	*Elderly donors*
*CASP3*	caspase 3	Cytoplasm	-750,876	-1461,671	-175,986
*COL1A1*	collagen type I alpha 1 chain	Extracellular Space	-7,947	-7,579	-2,429
*COL1A2*	collagen type I alpha 2 chain	Extracellular Space	-3,024	-4,831	18,091
*COL3A1*	collagen type III alpha 1 chain	Extracellular Space	-2,381	1,105	2,785
*COL4A1*	collagen type IV alpha 1 chain	Extracellular Space	1,004	-2,867	-1,087
*COL7A1*	collagen type VII alpha 1 chain	Extracellular Space	4,768	2,253	-1,302
*CXCL8*	C-X-C motif chemokine ligand 8	Extracellular Space	6,04	35,038	9,697
*IL10*	interleukin 10	Extracellular Space	76,565	113,444	108,814
*IL1A*	interleukin 1 alpha	Extracellular Space	-23,606	-6,03	-37120,195
*IL1B*	interleukin 1 beta	Extracellular Space	-16,093	-9,043	-382,792
*IL6*	interleukin 6	Extracellular Space	-5,87	-73,907	-2,008
*LMNA*	lamin A/C	Nucleus	1,433	-2,648	1,081
*MMP1*	matrix metallopeptidase 1	Extracellular Space	1,155	-9,11	-1,981
*MMP10*	matrix metallopeptidase 10	Extracellular Space	63,92	3,475	-38,757
*MMP12*	matrix metallopeptidase 12	Extracellular Space	6,859	8,168	8,685
*MMP13*	matrix metallopeptidase 13	Extracellular Space	1,045	6,621	5,632
*MMP14*	matrix metallopeptidase 14	Extracellular Space	2,914	1,863	1,321
*MMP2*	matrix metallopeptidase 2	Extracellular Space	-2,183	-60,805	1,032
*MMP3*	matrix metallopeptidase 3	Extracellular Space	8,251	41,26	-4,66
*MMP7*	matrix metallopeptidase 7	Extracellular Space	-53,59	-6,578	-461,894
*MMP8*	matrix metallopeptidase 8	Extracellular Space	-1,261	-5,08	-5,356
*MMP9*	matrix metallopeptidase 9	Extracellular Space	19173,227	-1,2	-1,566
*PTGS2*	prostaglandin-endoperoxide synthase 2	Cytoplasm	-9,872	-1,743	-4,534
*SIRT1*	sirtuin 1	Nucleus	-1,13	-3,221	3,186
*TIMP1*	TIMP metallopeptidase inhibitor 1	Extracellular Space	3,472	-1,523	2,258
*TIMP2*	TIMP metallopeptidase inhibitor 2	Extracellular Space	2,35	-2,218	1,807
*TIMP3*	TIMP metallopeptidase inhibitor 3	Extracellular Space	-11,337	-848,651	-1,766
*TIMP4*	TIMP metallopeptidase inhibitor 4	Extracellular Space	4,231	-1,314	-1,443
*TP53*	tumor protein p53	Nucleus	1,777	-5,406	-1,198

## Discussion

### Gene expression analysis

The RNA extraction represents a specific time for the condition analyzed. In these proposed aging models, the RNA extraction assay was performed 24 hours after stress stimulation. With regard to inflammatory pathway, we found *IL1B*, *IL1A*, *IL6*, *and PTGS2* downregulated gene expression, whereas *IL8* and *IL10* upregulated it. This result may suggest a regulation feedback caused by the proinflammatory cytokines in the cellular environment during the transcription–translation period. Studies have proven inflammation to be a physiological aging process that is associated with increased levels of cytokine circulation and proinflammatory markers [50]. The proinflammatory signaling, during our experiment, may have generated a feedback signal to the cells, inhibiting gene expression in order to decrease cytokine synthesis. These proinflammatory cytokines may also have generated a signal to stimulate the expression of the *IL10* gene, an anti-inflammatory cytokine. The cytokines involved in the inflammatory response indicate a possible mechanism to restore cell balance.

All genes from the inflammatory pathway considered in this study (*IL1B*, *IL1A*, *IL6*, *IL8*, *IL10*, *PTGS2*) showed consistency of aging influence on cell metabolism and functionality. In addition, inflammation is a common condition of the skin observed in the aging process [50; 51].

For *COL1A1*, all three in-vitro aging models showed a decrease of gene expression profile, indicating the direct influence of aging on collagen synthesis. The *COL1A2* gene was downregulated in both in-vitro aging models: HDF under UVB irradiation and accelerated proliferation. Whereas, it was upregulated in HDF from elderly donors, suggesting a different mechanism of collagen synthesis reduction during canonical skin aging. These genes, *COL1A1* and *COL1A2*, together encode the type I collagen protein, an abundant extracellular protein in the skin [52]. The *COL3A1* gene encodes the type III collagen, an important protein for skin balance together with collagen type I [53]. We found the downregulation of gene expression on HDF undergoing UVB irradiation but not in the HDF undergoing accelerated proliferation or HDF from elderly donors, which indicated that the synthesis of type III collagen is influenced by external environmental factors. The culmination of these findings provides strong evidence that our in-vitro skin aging model using UVB irradiation reproduces a realistic model for skin aging drug screening.

The matrix metalloproteinases are involved in the degradation of various proteins in the extracellular matrix [54] and plays an important role in the skin aging process. High levels of *MMP9* gene expression only in the HDF from UV radiation assay was observed. These findings are in agreement with the available literature [55], confirming the influence of UV radiation on inducing premature skin aging. Although *MMP9* presented a strong response to UVB radiation, other metalloproteinases were also upregulated, induced by the UVB radiation, such as *MMP1*, *MMP3*, *MMP10*, *MMP12*, *MMP13*, and *MMP14*. The *MMP12*, *MMP13*, and *MMP14* biosynthesis was upregulated in all three in-vitro aging models when compared with the young control, suggesting a crucial role of these genes in the skin aging biological mechanism. The tissue inhibitors of metalloproteinases *TIMP1*, *TIMP2*, and *TIMP4* were also upregulated during UVB irradiation, probably related to the increase in metalloproteinases expression. These findings suggest that the combination of *MMPs* and *TIMPs* gene expression analysis can be used as a biomarker for skin aging models to find new ingredients and prevent premature aging.

The *TP53* gene plays a central role in the regulation of cell division, inhibiting the uncontrolled cell proliferation behavior. The decrease of gene expression in the HDF from the accelerated proliferation assay and the HDF from elderly donors, but not in the HDF from UVB irradiation, indicates the senescence phenotype in these two models. The increasing of gene expression in that case may be considered a cell response to prevent any future tumor. This model can be a potential candidate for skin cancer evaluation.

*SIRT1*, an important biomarker in longevity studies, showed a decrease of gene expression in both in-vitro aging models, but not in the HDF from elderly donors. The biosynthesis downregulation of *SIRT1* observed in previous studies with UV radiation [56; 57] was observed here as well. These findings suggest that the use of UVB irradiation and the accelerated proliferation protocol are both realistic models that can support skin aging studies for this biomarker.

The *CASP3* was downregulated in all three in-vitro aging models evaluated, indicating that gene is an important biomarker for aging studies. *LMNA* is responsible for the synthesis of Lamin A and is produced in most cells of the body [58]. A mutation in the expression results in a truncated form of Lamin A, called progerin, whose accumulation has not only been described in HGPS but also during normal and photo-stimulated aging [31]. In this study, the LMNA was upregulated in the UVB irradiation model, indicating the strong influence of this gene in premature skin aging.

In contrast to studies which did not present any significant difference between the cells from in-vitro aging models [12; 59], in this study, the difference in the gene expression profile was observed when comparing HDF from young donors and the three in-vitro aging models [Table 1]. Comparing all in-vitro aging models of this study, 14 genes (*CASP3*, *COL1A1*, *MMP7*, *MMP8*, *MMP12*, *MMP13*, *MMP14*, *TIMP3*, *IL1B*, *IL1A*, *IL6*, *IL8*, *IL10*, *PTGS2*) showed similar gene expression behaviors. For 6 genes (*TP53*, *COL3A1*, *COL4A1*, *MMP1*, *MMP9*, *TIMP4*), different gene expression profiles were observed only in the cells from the UVB irradiation model, indicating major influence of the environment on these biomarkers. The HDF from elderly donors showed different gene expression profiles for 6 other genes (*SIRT1*, *COL1A2*, *COL7A1*, *MMP2*, *MMP3*, *MMP10*), and the HDF from the accelerated proliferation model showed the lowest number of genes with different gene expression profile: 3 genes (*LMNA*, *TIMP1*, *TIMP2*).

With regard to the use of the HDF from elderly donors, the gene expression response showed higher variability than previous findings in the literature suggest, when cultivated in vitro without aging signaling, thus exhibiting different behavior from fibroblasts of premature aging.

### Cell physiology and morphology

HDF may undergo changes in metabolism after natural aging or during excessive environmental exposure, resulting in visible signs of skin aging [6]. HDF from young and elderly donors as well as HDF from the assays of UVB irradiation and accelerated proliferation were compared in terms of cell growth rate in order to observe the influence of aging on cellular physiology and morphology, using an in-vitro model. The senescence phenotype in aged HDF was demonstrated through high levels of the β-galactosidase enzyme activity found in cells with blue coloration due to reaction with X-GAL [37]. Only HDF that presented aging activity was blue colored (Fig 1). The cell-counting assay reinforced the senescence-associated phenotype. The aged HDF decreased the cell growth rate in comparison to HDF from young donors (Fig 2). Initially, the results from ECIS revealed opposite result of cell-counting assay, exhibiting reduced doubling time of the aged HDF, through impedance sensing. However, it is known that aged HDF shows alterations in cell morphology with a larger cellular size than HDF from young donors [47; 48]; therefore, they showed reduced time to coat the well adapted ECIS (Fig 3) [45].

### Extrinsic aging model

The extrinsic skin aging process occurs in conjunction with intrinsic aging, associated with excessive environment exposition, resulting in visible signs of premature aging in an individual [20]. To observe the possible differences in pattern based on the cell aging process, the gene expression profile of HDF from young donors under UVB irradiation was analyzed. Both *COL1A1* and *COL1A2* were downregulated, indicating a decrease of collagen protein synthesis which is associated with premature aging. The increase in *MMP1*, *MMP3*, *MMP9*, *MMP10*, *MMP12*, *MMP13*, *and MMP14* in addition to *TIMP1*, *TIMP2*, *and TIMP4* gene expressions were observed during the experiment, supporting the influence of the UVB stimulus in premature skin aging signaling [21]. *LMNA* and *TP53* were also upregulated, corroborating the findings from the literature on the relationship between the accumulation of these proteins and aging [31].

### Intrinsic aging model

The process of intrinsic aging occurs over the lifespan, leading to a decrease of cell functions and the beginning of the senescence process [60]. Several biological processes are involved in this effect, but the damage in telomeres caused by the cellular replication process is the main factor [61].

In this study, HDF from young healthy donors were cultivated until passage 20, senescence activity, and a decrease in cell growth rate were observed. To observe the differences in the aging process through accelerated proliferation, we evaluated the gene expression profiles of these cells. The *COL1A1*, *COL1A2*, *and COL4A1* genes were downregulated, indicating a decrease in collagen synthesis related to aging. The *MMP1*, *MMP2*, *MMP7*, *MMP8*, *and MMP9* as well as the *TIMP1*, *TIMP2*, *TIMP3 and TIMP4* genes were downregulated in this model, contrasting with the UVB irradiation aging model and demonstrating the necessity for an external signaling to induce alterations in such cellular physiological processes in vitro [19].

### HDF from elderly donor

The collagen gene expression from HDF progressively decreases, and the enzymes responsible for collagen degradation increase with age. Other biological mechanisms, such as inflammation and apoptosis pathway, also induce alterations in normal metabolism. In this study, we found that the *COL1A1*, *COL4A1*, *and COL7A1* gene expressions decreased in HDF from elderly donors, corroborating the findings in the relevant literature. The genes *MMP1*, *MMP3*, *MMP7*, *MMP8*, *MMP9*, *and MMP10* were downregulated, suggesting that an external inducing factor is necessary for this stimulation.

## Conclusion

Results from all three in-vitro aging methodologies was successful in proving senescence activity. Regarding the gene expression profile, we found 14 genes with similar gene expression behaviors in all three in-vitro aging models in addition to the genes with particular behavior pertaining to differences in the applied stress model, such UVB irradiation or accelerated proliferation. In this study, we demonstrated the existence of a gene expression pattern in aging. Furthermore, we also demonstrated the viability of using these in-vitro methodologies for aging studies’ models or drug discovery screening as well as the use of these genes as biological aging markers.

## Supporting information

S1 File Scepter cell counter assay. Cell proliferation time technique performed according Scepter Cell Counter protocol.
(XLSX)

S2 File ECIS assay. Real-time cell proliferation analysis performed according to the ECIS protocol.
(XLSX)

S3 File Gene expression analysis. Gene expression levels were estimated using the ΔΔCt methodology and keeping HDF from young donors as a control.
(XLSX)

S4 File Statistic analysis. The statistical significance between the three in-vitro models groups using the ΔΔCt results from the real-time PCR assay was obtained by performing ANOVA in the XLSTAT 2007 program. For the Scepter Cell Counter assay, this was analyzed by Test Z, XLSTAT 2007 program.
(XLSX)

## References

[1] Thiele JJ, Podda M, Packer L. Tropospheric ozone: an emerging environmental stress to skin. *Biol Chem*. 1997, Vols. 378(11):1299–305. 10.1515/bchm.1997.378.11.1299 9426190

[2] Lephart ED. A review of the role of estrogen in dermal aging and facial attractiveness in women. *J Cosmet Dermatol*. 10.1111/jocd.12508, 2018 29436770

[3] Ibuki A, Kuriyama S, Toyosaki Y, Aiba M, Hidaka M, Horie Y, Fujimoto C, Isami F, Shibata E, Terauchi Y, Akase T. Aging-like physiological changes in the skin of Japanese obese diabetic patients. *SAGE Open Med*. 10.1177/2050312118756662, 2018, Vol. 6:2050312118756662. 29449943 PMC5808963

[4] Dyer JM, Miller RA. Chronic Skin Fragility of Aging: Current Concepts in the Pathogenesis, Recognition, and Management of Dermatoporosis. *J Clin Aesthet Dermatol*.. 2018, Vols. 11(1):13–18. PMC5788262 29410724

[5] Farage MA, Miller KW, Berardesca E, Maibach HI. Clinical implications of aging skin: cutaneous disorders in the elderly. *Am J Clin Dermatol*. 10.2165/00128071-200910020-00001, 2009, Vols. 10(2):73–86. 19222248

[6] Vierkötter A, Krutmann J. Environmental influences on skin aging and ethnic-specific manifestations. *Dermatoendocrinol*. 10.4161/derm.19858, 2012, Vols. 1;4(3):227–31. 23467702 PMC3583881

[7] Slominsk AT, Manna PR, Tuckey RC,. On the role of skin in the regulation of local and systemic steroidogenic activities. *Steroids*. 103: 72–88, 2015 10.1016/j.steroids.2015.04.006 25988614 PMC4631694

[8] Tobin DJ. Introduction to skin aging. *J Tissue Viability*. 26(1):37–46, 2017 10.1016/j.jtv.2016.03.002 27020864

[9] Hatem S, Nasr M, Elkheshen SA, Geneidi AS. Recent advances in antioxidant cosmeceutical topical delivery. *Curr Drug Deliv*. 10.2174/1567201815666180214143551, 2018 29446743

[10] Ekanayake Mudiyanselage S, Hamburger M, Elsner P, Thiele JJ. Ultraviolet a induces generation of squalene monohydroperoxide isomers in human sebum and skin surface lipids in vitro and in vivo. *J Invest Dermatol*. 2003, Vols. 120(6):915–22. 10.1046/j.1523-1747.2003.12233.x 12787115

[11] Slominski A, Wortsman J. Neuroendocrinology of the skin. *Endocr Rev*. 21(5):457–87, 2000 10.1210/edrv.21.5.0410 11041445

[12] Kaisers W, Boukamp P, Stark HJ, Schwender H, Tigges J, Krutmann J, Schaal H. Age, gender and UV-exposition related effects on gene expression in in vivo aged short term cultivated human dermal fibroblasts. *PLoS One*. 10.1371/journal.pone.0175657, 2017, Vol. 12(5):e0175657 28475575 PMC5419556

[13] Chen H, Wang X, Han J, Fan Z, Sadia S, Zhang R, Guo Y, Jiang Y, Wu Y. AKT and its related molecular feature in aged mice skin. *PLoS One*. 10.1371/journal.pone.0178969, 2017, Vol. 12(6):e0178969 28591208 PMC5462418

[14] Shoko T, Maharaj VJ, Naidoo D, Tselanyane M, Nthambeleni R, Khorombi E, Apostolides Z. Anti-aging potential of extracts from Sclerocarya birrea (A. Rich.) Hochst and its chemical profiling by UPLC-Q-TOF-MS. *BMC Complement Altern Med*. 10.1186/s12906-018-2112-1, 2018, Vol. 18(1):54 29415712 PMC5804067

[15] Na J, Bak DH, Im SI, Choi H, Hwang JH, Kong SY, No YA, Lee Y, Kim BJ. Anti-apoptotic effects of glycosaminoglycans via inhibition of ERK/AP-1 signaling in TNF-α-stimulated human dermal fibroblasts. *Int J Mol Med*. 10.3892/ijmm.2018.3483, 2018. 29436595

[16] Li L, Hwang E, Ngo HTT, Lin P, Gao W, Liu Y, Yi TH. Anti-photoaging Effect of Prunus yeonesis Blossom Extract via Inhibition of MAPK/AP-1 and Regulation of the TGF-βI/Smad and Nrf2/ARE Signaling Pathways. *Photochem Photobiol*. 10.1111/php.12894, 2018. 29421853

[17] Tamura E, Ishikawa J, Sugata K, Tsukahara K, Yasumori H, Yamamoto T. Age-related differences in the functional properties of lips. *Skin Res Technol*. 10.1111/srt.12456, 2018 29405429

[18] Porcheron A, Mauger E, Russell R. Aspects of facial contrast decrease with age and are cues for age perception. *PLoS One*. 10.1371/journal.pone.0057985, 2013, Vol. 8(3):e57985. 23483959 PMC3590275

[19] Bai B, Liu Y, You Y, Li Y, Ma L. Intraperitoneally administered biliverdin protects against UVB-induced skin photo-damage in hairless mice. *J Photochem Photobiol B*. 10.1016/j.jphotobiol.2015.02.001, 2015, Vols. 144:35–41. 25689514

[20] Quan T, Qin Z, Xia W, Shao Y, Voorhees JJ, Fisher GJ. Matrix-degrading metalloproteinases in photoaging. *J Investig Dermatol Symp Proc*. 10.1038/jidsymp.2009.8, 2009, Vols. 14(1):20–4. 19675548 PMC2909639

[21] Fligiel SE, Varani J, Datta SC, Kang S, Fisher GJ, Voorhees JJ. Collagen degradation in aged/photodamaged skin in vivo and after exposure to matrix metalloproteinase-1 in vitro. *J Invest Dermatol*.. 2003, Vols. 120(5):842–8. 10.1046/j.1523-1747.2003.12148.x 12713591

[22] Varani J, Dame MK, Rittie L, Fligiel SE, Kang S, Fisher GJ, Voorhees JJ. Decreased collagen production in chronologically aged skin: roles of age-dependent alteration in fibroblast function and defective mechanical stimulation. *Am J Pathol*.. 2006, Vols. 168(6):1861–8. 10.2353/ajpath.2006.051302 PMC1606623 16723701

[23] Aoki M, Miyake k, Ogawa R, Dohi T, Akaishi S, Hyakusoku H, Shimada T. siRNA Knockdown of Tissue Inhibitor of Metalloproteinase-1 in Keloid Fibroblasts Leads to Degradation of Collagen Type I. *Journal of Investigative Dermatology*. 10.1038/jid.2013.396, 2014, Vols. 134, 818–826. 24042342

[24] Ulrich D, Ulrich F, Unglaub F, Piatkowski A, Pallua N. Matrix metalloproteinases and tissue inhibitors of metalloproteinases in patients with different types of scars and keloids. *J Plast Reconstr Aesthet Surg*. 10.1016/j.bjps.2009.04.021, 2010, Vols. 63(6):1015–21. 19464975

[25] Abbas H, Kamel R, El-Sayed N. Dermal anti-oxidant, anti-inflammatory and anti-aging effects of Compritol ATO-based Resveratrol colloidal carriers prepared using mixed surfactants. *Int J Pharm*.. 10.1016/j.ijpharm.2018.01.054, 2018, Vols. 29458209

[26] Amar SK, Goyal S, Mujtaba SF, Dwivedi A, Kushwaha HN, Verma A, Chopra D, Chaturvedi RK, Ray RS. Role of type I & type II reactions in DNA damage and activation of caspase 3 via mitochondrial pathway induced by photosensitized benzophenone. *Toxicol Lett*. 10.1016/j.toxlet.2015.03.008, 2015, Vols. 235(2):84–95. 25800561

[27] Subedi L, Lee TH, Wahedi HM, Baek SH, Kim SY. Resveratrol-Enriched Rice Attenuates UVB-ROS-Induced Skin Aging via Downregulation of Inflammatory Cascades. *Oxid Med Cell Longev*. 10.1155/2017/8379539, 2017, Vol. 2017:8379539. 28900534 PMC5576414

[28] Kilic U, Gok O, Erenberk U, Dundaroz MR, Torun E, Kucukardali Y, Elibol-Can B, Uysal O, Dundar T. A remarkable age-related increase in SIRT1 protein expression against oxidative stress in elderly: SIRT1 gene variants and longevity in human. *PLoS One*. 10.1371/journal.pone.0117954, 2015, Vol. 18;10(3):e0117954 25785999 PMC4365019

[29] McKenna T, Sola Carvajal A, Eriksson M. Skin Disease in Laminopathy-Associated Premature Aging. *J Invest Dermatol*.. 10.1038/jid.2015.295, 2015, Vols. 135(11):2577–2583. 26290387

[30] DeBoy E, Puttaraju M, Jailwala P, Kasoji M, Cam M, Misteli T. Identification of novel RNA isoforms of LMNA. *Nucleus*. 10.1080/19491034.2017.1348449, 2017, Vols. 3;8(5):573–582. 28857661 PMC5703264

[31] Takeuchi H, Rünger TM. Longwave UV light induces the aging-associated progerin. *J Invest Dermatol*. 10.1038/jid.2013.71, 2013, Vols. 133(7):1857–62. 23392295

[32] Souto LR, Rehder J, Vassallo J, Cintra ML, Kraemer MH, Puzzi MB. Model for human skin reconstructed in vitro composed of associated dermis and epidermis. *Sao Paulo Med J*. 124(2):71–6, 2006 16878189 10.1590/S1516-31802006000200005 PMC11060353

[33] Souto LR, Vassallo J, Rehder J, Pinto GA, Puzzi MB,. Immunoarchitectural characterization of a human skin model reconstructed in vitro. *Sao Paulo Med J*. 127(1):28–33, 2009 19466292 10.1590/S1516-31802009000100007 PMC10969314

[34] Straface E, Vona R, Ascione B, Matarrese P, Strudthoff T, Franconi F, Malorni W. Single exposure of human fibroblasts (WI-38) to a sub-cytotoxic dose of UVB induces premature senescence. *FEBS Letters*. 581(22):4342–8, 2007 10.1016/j.febslet.2007.08.006 17716665

[35] Briske-Anderson MJ, Finley JW, Newman SM. The influence of culture time and passage number on the morphological and physiological development of Caco-2 cells. *Proc Soc Exp Biol Med*. 214(3):248–57, 1997 10.3181/00379727-214-44093 9083258

[36] Chang-Liu CM, Woloschak GE. Effect of passage number on cellular response to DNA-damaging agents: cell survival and gene expression. *Cancer Lett*. 113(1–2):77–86, 1997 9065805 10.1016/s0304-3835(97)04599-0

[37] Gary RK, Kindell SM. Quantitative assay of senescence-associated beta-galactosidase activity in mammalian cell extracts. *Anal Biochem*.. 2005, Vols. 343(2):329–34. 10.1016/j.ab.2005.06.003 16004951

[38] Applied BioPhysics, Inc. [Online] http://www.biophysics.com/PDFS.php?source=products.

[39] Qiagen. RNeasy Mini Kit: For purification of total RNA from animal cells, animal tissues, bacteria, and yeast, and for RNA cleanup *RNeasy*^*^®^*^*Mini Handbook*. 2012.

[40] Livak KJ, Schmittgen TD. Analysis of relative gene expression data using real-time quantitative PCR and the 2(-Delta Delta C(T)) Method. *Methods*. 25(4):402–8, 2001 10.1006/meth.2001.1262 11846609

[41] Hayflick L. The Limited In Vitro Lifetime Of Human Diploid Cell Strains. *Exp Cell Res*. 1965, Vols. 37:614–36. 10.1016/0014-4827(65)90211-9 14315085

[42] Jobeili L, Rousselle P, Béal D, Blouin E, Roussel AM, Damour O, Rachidi W. Selenium preserves keratinocyte stemness and delays senescence by maintaining epidermal adhesion. *Aging*. 10.18632/aging.101322, 2017, Vols. 25;9(11):2302–2315. 29176034 PMC5723688

[43] Park JT, Kang HT, Park CH, Lee YS, Cho KA, Park SC. A crucial role of ROCK for alleviation of senescence-associated phenotype. *Exp Gerontol*.. 10.1016/j.exger.2018.02.012, 2018, Vols. 29474864

[44] Pannese E. Morphological changes in nerve cells during normal aging. *Brain Struct Funct*. 10.1007/s00429-011-0308-y, 2011, Vols. 216(2):85–9. 21431333

[45] Giaever I, Keese CR. Monitoring fibroblast behavior in tissue culture with an applied electric field. *PNAS*. 1984, Vols. 81, pp. 3761–3764. 10.1073/pnas.81.12.3761 PMC345299 6587391

[46] Giaever I, Keese CR. Use of electric fields to monitor the dynamical aspect of cell behavior in tissue culture. *IEEE Trans*. *Biomed*. *Eng*. 1986, Vols. 33, pp. 242–247. 10.1109/TBME.1986.325896 3957373

[47] Yang J, Dungrawala H, Hua H, Manukyan A, Abraham L, Lane W, Mead H, Wright J, Schneider BL. Cell size and growth rate are major determinants of replicative lifespan. *Cell Cycle*. 10.4161/cc.10.1.14455, 2011, Vols. 10(1):144–55. 21248481 PMC3048081

[48] Biran A, Zada L, Abou Karam P, Vadai E, Roitman L, Ovadya Y, Porat Z, Krizhanovsky V. Quantitative identification of senescent cells in aging and disease. *Aging Cell*. 10.1111/acel.12592, 2017, Vols. 16(4):661–671. 28455874 PMC5506427

[49] Durai PC, Thappa DM, Kumari R, Malathi M. Aging in elderly: chronological versus photoaging. *Indian J Dermatol*. 10.4103/0019-5154.100473, 2012, Vols. 57(5):343–52. 23112352 PMC3482795

[50] Michaud M, Balardy L, Moulis G, Gaudin C, Peyrot C, Vellas B, Cesari M, Nourhashemi F. Proinflammatory cytokines, aging, and age-related diseases. *J Am Med Dir Assoc*. 10.1016/j.jamda.2013.05.009, 2013, Vols. 14(12):877–82. 23792036

[51] Kostyuk V, Potapovich A, Stancato A, De Luca C, Lulli D, Pastore S, Korkina L. Photo-oxidation products of skin surface squalene mediate metabolic and inflammatory responses to solar UV in human keratinocytes. *PLoS One*. 10.1371/journal.pone.0044472, 2012, Vol. 7(8):e44472 22952984 PMC3431355

[52] Sharma U, Carrique L, Vadon-Le Goff S, Mariano N, Georges RN, Delolme F, Koivunen P, Myllyharju J, Moali C, Aghajari N, Hulmes DJ. Structural basis of homo- and heterotrimerization of collagen I. *Nat Commun*. 8:14671, 2017. 10.1038/ncomms14671 PMC5353611 28281531

[53] Remoué N, Molinari J, Andres E, Lago JC, Barrichello C, Moreira PL. Development of an in vitro model of menopause using primary human dermal fibroblasts. *Int J Cosmet Sci*. 35(6):546–54, 2013 10.1111/ics.12075 23802717

[54] Cui N, Hu M, Khalil RA. Biochemical and Biological Attributes of Matrix Metalloproteinases. *Prog Mol Biol Transl Sci*. 147:1–73, 2017 10.1016/bs.pmbts.2017.02.005 28413025 PMC5430303

[55] de Souza RO, de Assis Dias Alves G, Aguillera ALS, Rogez H, Fonseca MJV. Photochemoprotective effect of a fraction of a partially purified extract of Byrsonima crassifolia leaves against UVB-induced oxidative stress in fibroblasts and hairless mice. *J Photochem Photobiol B*. 178:53–60, 2018 10.1016/j.jphotobiol.2017.10.033 29107927

[56] Chung KW, Choi YJ, Park MH, Jang EJ, Kim DH, Park BH, Yu BP, Chung HY. Molecular Insights into SIRT1 Protection Against UVB-Induced Skin Fibroblast Senescence by Suppression of Oxidative Stress and p53 Acetylation. *J Gerontol A Biol Sci Med Sci*. 10.1093/gerona/glu137, 2015, Vols. 70(8):959–68. 25165029

[57] Ming M, Soltani K, Shea CR, Li X, He YY. Dual role of SIRT1 in UVB-induced skin tumorigenesis. *Oncogene*. 10.1038/onc.2013.583, 2015, Vols. 34(3):357–63. 24441046 PMC4104262

[58] National Institutes of Health. Genetics Home Reference. [Online] [Cited: Feb 2018, 27.] https://ghr.nlm.nih.gov/gene/LMNA.

[59] Boraldi F, Annovi G, Tiozzo R, Sommer P, Quaglino D. Comparison of ex vivo and in vitro human fibroblast ageing models. *Mech Ageing Dev*. 10.1016/j.mad.2010.08.008, 2010, Vols. 131(10):625–35. 20816692

[60] Dimri GP, Lee X, Basile G, Acosta M, Scott G, Roskelley C, Medrano EE, Linskens M, Rubelj I, Pereira-Smith O, et al A biomarker that identifies senescent human cells in culture and in aging skin in vivo. *PNAS*. 1995, Vols. 92(20):9363–7. 10.1073/pnas.92.20.9363 PMC40985 7568133

[61] Aubert G, Lansdorp PM. Telomeres and aging. *Physiol Rev*. 10.1152/physrev.00026.2007, 2008, Vols. 88(2):557–79. 18391173

